# *De novo* Transcriptome Analysis of *Miscanthus lutarioriparius* Identifies Candidate Genes in Rhizome Development

**DOI:** 10.3389/fpls.2017.00492

**Published:** 2017-04-12

**Authors:** Ruibo Hu, Changjiang Yu, Xiaoyu Wang, Chunlin Jia, Shengqiang Pei, Kang He, Guo He, Yingzhen Kong, Gongke Zhou

**Affiliations:** ^1^Key Laboratory of Biofuels, Qingdao Engineering Research Center of Biomass Resources and Environment, Qingdao Institute of Bioenergy and Bioprocess Technology, Chinese Academy of SciencesQingdao, China; ^2^Shandong Institute of Agricultural Sustainable DevelopmentJinan, China; ^3^Key Laboratory of Tobacco Genetic Improvement and Biotechnology, Tobacco Research Institute of Chinese Academy of Agricultural SciencesQingdao, China

**Keywords:** *Miscanthus lutarioriparius*, rhizome formation, RNA-Seq, lateral meristem, transcription factor

## Abstract

**HIGHLIGHT**

- *De novo* transcriptome profiling of five tissues reveals candidate genes putatively involved in rhizome development in *M. lutarioriparius*.

*Miscanthus lutarioriparius* is a promising lignocellulosic feedstock for second-generation bioethanol production. However, the genomic resource for this species is relatively limited thus hampers our understanding of the molecular mechanisms underlying many important biological processes. In this study, we performed the first *de novo* transcriptome analysis of five tissues (leaf, stem, root, lateral bud and rhizome bud) of *M. lutarioriparius* with an emphasis to identify putative genes involved in rhizome development. Approximately 66 gigabase (GB) paired-end clean reads were obtained and assembled into 169,064 unigenes with an average length of 759 bp. Among these unigenes, 103,899 (61.5%) were annotated in seven public protein databases. Differential gene expression profiling analysis revealed that 4,609, 3,188, 1,679, 1,218, and 1,077 genes were predominantly expressed in root, leaf, stem, lateral bud, and rhizome bud, respectively. Their expression patterns were further classified into 12 distinct clusters. Pathway enrichment analysis revealed that genes predominantly expressed in rhizome bud were mainly involved in primary metabolism and hormone signaling and transduction pathways. Noteworthy, 19 transcription factors (TFs) and 16 hormone signaling pathway-related genes were identified to be predominantly expressed in rhizome bud compared with the other tissues, suggesting putative roles in rhizome formation and development. In addition, a predictive regulatory network was constructed between four TFs and six auxin and abscisic acid (ABA) -related genes. Furthermore, the expression of 24 rhizome-specific genes was further validated by quantitative real-time RT-PCR (qRT-PCR) analysis. Taken together, this study provide a global portrait of gene expression across five different tissues and reveal preliminary insights into rhizome growth and development. The data presented will contribute to our understanding of the molecular mechanisms underlying rhizome development in *M. lutarioriparius* and remarkably enrich the genomic resources of *Miscanthus*.

## Introduction

Currently, global climate change and depleting fossil fuel reserves have spurred increased concerns in the pursuit of alternative renewable energy resources such as biofuels (Yuan et al., 2008). *Miscanthus* is a temperate perennial C4 grass that belongs to the Andropogoneae tribe, which consists of many economically important crops including maize, sorghum, and sugarcane. *Miscanthus* has been regarded as a promising bioenergy crop for lignocellulosic biofuel production due to its superior characteristics such as high biomass yield, perennial growth habitus, low water and fertilizer requirement, abiotic stress tolerance and broad adaptation to marginal land etc. (Lewandowski et al., 2000; Parveen et al., 2013; Robson et al., 2013; Lee and Kuan, 2015; Xing et al., 2016). The *Miscanthus* genus contains more than 14 species, most of which originate from southern and eastern Asia (Hodkinson et al., 2002). Among these species, *Miscanthus lutarioriparius* that is endemic to central China, is characterized by overwhelmingly higher biomass yield and outstanding restoration capability of marginal land compared with *M. sacchariflorus* and *M. sinensis* (Liu et al., 2012; Yan et al., 2012; Mi et al., 2014). Thus, *M. lutarioriparius* is currently the most widely cultivated species for biomass production in bioenergy and biorefinery in China (Mi et al., 2014).

In particular, one of the most striking characteristics of *M. lutarioriparius* beyond its high productivity, is its conspicuous rhizome compared to other *Miscanthus* species. Rhizome cutting is the primary means of propagation for *M. lutarioriparius* because of its self-incompatibility. Rhizomes are modified subterranean stems, which have both apical and lateral meristems capable of generating adventitious shoots and roots (Yoshida et al., 2016). Rhizomes play important roles in the persistence of many perennial grass species, serving as the primary reservoir for energy storage and propagation organ (Jernstedt and Bouton, 1985; Li and Beuselinck, 1996; Glover et al., 2010). To some extent, rhizomatous growth is a desirable attribute in the establishment and persistence to various abiotic stresses for plants cultivated in adverse growth conditions (e.g., marginal land) (Sacks et al., 2006; Su et al., 2008; Zhou et al., 2014). However, because of the advantages of vigorous propagation, rhizomatous growth is also adopted by some of the most noxious weeds to grow vigorously and rapidly by strong rhizomes, which renders them to invade a wide range of regions and has raised increasing ecological concerns worldwide (Paterson et al., 1995).

Despite of the important roles of rhizome in plant growth and development, the genetic and molecular mechanisms underlying rhizome initiation and growth remain largely unknown. Rhizome development is a very complicated process, which is synergistically regulated by both intrinsic cues and environmental stimuli. Recently, with the advent of next-generation sequencing (NGS) technology, high-throughput RNA sequencing (RNA-seq) has become a powerful and cost-efficient means to discover putative functional genes involved in diverse biological processes, especially for plant species without a reference genome. RNA-seq has also been employed to identify genes related to rhizome development in various rhizomatous species including *Oryza longistaminata* (Hu et al., 2011), Sorghum (*Sorghum halepense* and *Sorghum propinquum*) (Jang et al., 2006; Zhang et al., 2014), reed (*Phragmites australis*) (He et al., 2012), bamboo (*Phyllostachys praecox*) (Wang et al., 2010), lotus (*Nelumbo nucifera*) (Cheng et al., 2013; Yang et al., 2015), Cangzhu (*Atractylodes lancea*) (Huang et al., 2016), Ginger (*Zingiber officinale*) and turmeric (*Curcuma longa*) (Koo et al., 2013) etc. In addition, transcriptome profiling of genes related to the rejuvenation of spring rhizomes in *M*. × *giganteus* and the rhizome-specific genes in *M. sacchariflorus* has also been carried out (Barling et al., 2013; Kim et al., 2014). For instance, several TFs belonging to YABBY, TCP and homeobox families were identified to be specifically expressed in rhizome tips of *Oryza longistaminata*, suggesting putative roles in rhizome development (Hu et al., 2011). *PpHB1*, a homolog of Homeodomain-leucine Zipper (HD-ZIP) transcription factor (TF), was implicated to be responsible for procambial development and rhizome bud formation in bamboo (*P. praecox*) (Wang et al., 2010). The rhizome morphogenesis of lotus (*N. nucifera*) was revealed to be mediated by photoperiod as well as light spectra (Masuda et al., 2007). Twenty-two genes associated with pathways in photoperiod, starch metabolism and hormone signaling were preferentially expressed in lotus rhizome, indicating possible roles in rhizome development (Yang et al., 2015). These genes provided excellent candidates for further functional characterization to unravel their roles in rhizome differentiation, growth and development.

Although *M. lutarioriparius* has been considered as an important feedstock resource for biofuel and biorefinery, the genetic resources especially transcriptome data are currently very limited, which significantly restricts the genetic improvement of this species for desirable traits. Previously, transcriptome profiling has only been reported for genes related to water-use efficiency and high photosynthesis efficiency for *M. lutarioriparius* (Fan et al., 2015; Xing et al., 2016). Rhizomnousness is one of the most distinctive characteristics of *M. lutarioriparius* compared to the other *Miscanthus* species. Rhizome cutting is the primary means of propagation for *M. lutarioriparius* in biofuel production. Elucidating the molecular mechanisms underlying rhizome initiation and development is of fundamental importance, because it will not only contribute to our better understanding of this important biological process, but also serves as theoretical basis for rapid and efficient propagation of this species through rhizome cutting. However, the transcriptome profiling of genes involved in rhizome development has not been addressed thus far in *M. lutarioriparius*.

In this study, we performed deep transcriptome sequencing using an Illumina HiSeq 2500 platform to characterize gene expression in rhizomes and other four different tissues of *M. lutarioriparius*. To the best of our knowledge, this is the first report on the transcriptome profiling of candidate genes related to rhizome development in *M. lutarioriparius*. These findings could contribute to our better understanding of the molecular mechanisms underlying rhizome initiation and development in *M. lutarioriparius*. In addition, the data generated also present as valuable resources for further functional genomics research in *Miscanthus* species.

## Materials and methods

### Plant materials

*M. lutarioriparius* was propagated asexually via rhizomes from one plant collected from Changsha, Hu'nan province, China. Plants were grown in a growth chamber under 16 h light/ 8 h darkness photoperiod at 25°C. Leaves (fully expanded, upper apex), roots, stems (2nd top internode), rhizome buds (~3 cm) and shoot lateral buds (4th basal internode) were separately collected from 1-year-old potted plants. Each tissue has two replicates from six individual plants. All samples were immediately frozen in liquid nitrogen and stored at −80°C until use.

### RNA isolation

Total RNA was isolated using Trizol reagent (Invitrogen) according to the manufacturer's instructions. The extracted RNA was treated with DNase I (Promega) to remove the contaminated DNA. The quality of RNA was initially evaluated by electrophoresis in 1.5% agar gel, then quantified using the NanoDrop2000 spectrophotometer (ThermoFisher Scientific) and Agilent 2100 Bioanalyzer (Agilent Technologies). Only samples with the RNA integrity number (RIN) values higher than 8.0 were subjected to further analysis. Two biological replicates were used for RNA extraction and further transcriptome sequencing.

### RNA sequencing and *de novo* assembly

Ten cDNA libraries (two for each tissue) were constructed from purified mRNA using a NEBNext Ultra RNA Library Prep Kit following the manufacturer's protocols. The cDNA libraries were sequenced using the Illumina Hiseq 2500 platform by Novogene Company (Beijing, China). Raw reads obtained after sequencing were filtered by removing adapter containing reads, empty reads, and low quality reads (*Q* < 20). The remaining clean reads were *de novo* assembled into non-redundant unigenes using the Trinity program (Grabherr et al., 2011).

### Functional annotation of unigenes

Assembled unigenes were annotated using BLAST alignment against public databases, including the NCBI non-redundant protein database (NR), NCBI non-redundant nucleotide database (Nt), Swiss-Prot, Protein Family (Pfam), Gene Ontology (GO), eukaryotic Orthologous Groups (KOG) database, the Kyoto Encyclopedia of Genes and Genomes (KEGG), and KEGG Ortholog database (KO) with E-value threshold of 10^−5^.

### Analysis of differential expression genes (DEGs)

The expression level of each unigene was normalized and calculated as the value of fragments per kilobase of exon per million fragments mapped (FPKM) using the edgeR package (Robinson et al., 2010; Trapnell et al., 2010). The differentially expressed genes (DEGs) were determined by the criterion of fold change ≥2.0 and False Discovery Rate (FDR) ≤ 0.05. The tissue-enriched genes were defined as FPKM value is larger than 10 and the fold change of relative expression is higher than 3.0 compared to other tissues. Venn graph was drawn using an online tool (http://bioinformatics.psb.ugent.be/webtools/Venn/).

### Heatmap plotting of DEGs

Heatmap was generated with Z-score normalized FPKM values of the DEGs using the online OmicShare tools (www.omicshare.com/tools). The K-means clustering was conducted based on Pearson correlation of gene expression profiles.

### GO and KEGG enrichment analysis

The GO enrichment analysis of DEGs was implemented by the GOseq R packages based on Wallenius non-central hypergeometric distribution (Young et al., 2010). The KEGG pathway enrichment analysis of DEGs was performed using KOBAS software (Wu et al., 2006). Both analysis was tested at a significance cutoff of FDR ≤ 0.05.

### Identification of transcription factors (TFs)

The TF families were identified by BLASTX against known plant TFs identified in PlnTFDB database (http://plntfdb.bio.uni-potsdam.de/v3.0) with E-value threshold ≤ 10^−5^.

### Regulatory network construction

TFs and hormone signaling genes preferentially expressed in rhizome buds were selected for the generation of regulatory network. The Pearson coefficient was calculated between any two genes based on their FPKM values from five different tissues. A threshold of coefficient higher than 0.85 was adopted to discriminate significant co-expression between gene pairs. The network was displayed using Cytoscape software (Maere et al., 2005).

### Quantitative real-time RT-PCR (qRT-PCR) analysis

Twenty four unigenes were selected for the expression verification by qRT-PCR analysis. All reactions were carried out in 96-well plates in the LightCycler 480 detection system (Roche) using the SYBR Premix Ex Taq II (TaKaRa) kit. Amplification procedure is 95°C for 30 s, followed by 40 cycles of 95°C for 5 s, 60°C for 15 s, and 72°C for 10 s. The expression was normalized using reference gene *ACTIN11* and determined by the 2-deltadelta Ct method (Livak and Schmittgen, 2001). All reactions were performed with three replicates. All primers used are shown in Table S1.

## Results

### Sequencing and *de novo* assembly

To gain a comprehensive overview of *M. lutarioriparius* transcriptome, 10 cDNA libraries from leaf, stem, root, lateral bud and rhizome bud were constructed and sequenced using Illumina HiSeq 2500 platform. After filtering out adaptor sequences, ambiguous and low-quality reads, ~6.5 gigabase (GB) clean reads were acquired for each sample. The total clean reads summed to 66 GB for all samples (Table S2). Using the Trinity program, all clean reads were *de novo* assembled into 308,155 transcripts with an average length of 1,080 bp and an N50 length of 1,843 bp. These transcripts were further assembled into 169,064 unigenes. The size of the unigenes ranged from 201 to 17,395 bp, with a mean length of 759 bp and an N50 value of 1,339 bp. Among these unigenes, 35,599 (21.1%) were longer than 1,000 bp, and 100,775 (59.6%) were shorter than 500 bp (Figure S1).

### Functional annotation and classification

To perform annotation of the assembled unigenes, BLAST search was carried out against seven public protein/nucleotide databases with an E-value threshold of 10^−5^. In total, 103,899 (61.5%) unigenes were successfully annotated in at least one of these seven databases, while ~38.5% unigenes remain unmapped. The detailed gene annotation info was provided as Table S3.

**Figure 1:**
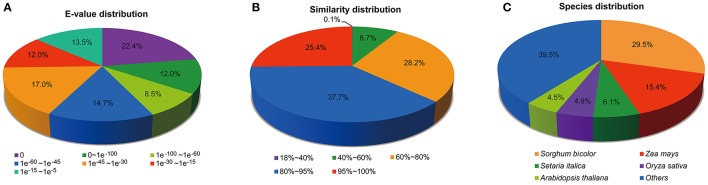
**Distribution of unigenes annotated to the NCBI NR protein database. (A)** E-value distribution of top BLAST hits against the NR database for each unigene. **(B)** Similarity distribution of top BLAST hits for each unigene. **(C)** Species distribution of top BLAST hits for matched unigene sequences.

According to the E-value distribution of significant hits against the NR database, 57.5% of the matched sequences showed significant homology with E-values less than 1.0E^−45^, while 42.5% of the mapped hit showed moderate homology with E-values between 1.0E^−45^ and 1.0E^−5^ (Figure 1A). In terms of similarity distribution, 63.1% of the matched sequences had a similarity higher than 80%, while 26.9% of the hits showed a similarity ranging from 18 to 80% (Figure 1B). Further analysis of homologies among different plant species revealed that the annotated unigenes had the highest homology with sequences from *Sorghum biocolor* (29.5%), followed by *Zea mays* (15.4%) and *Setaria italic* (6.1%) (Figure 1C). The top two species with the highest hits match are from the Poaceae family, indicating that the assembly and annotation of *M. lutarioriparius* transcriptome is proper and reliable.

**Figure 2:**
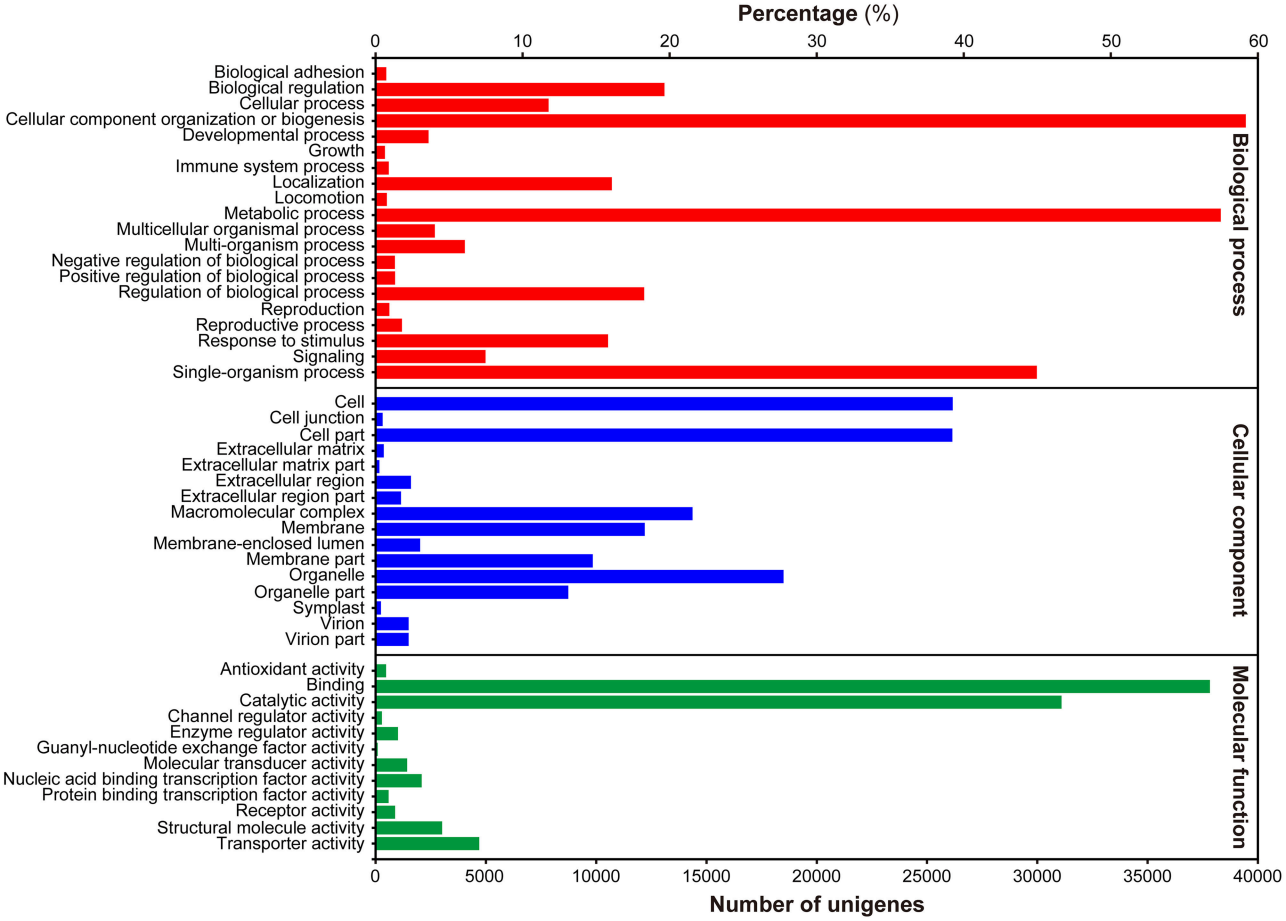
**Histogram presentation of GO classification**. Bars represent the numbers of unigenes matched to each GO term of three categories: Biological process (red), Cellular component (blue), and Molecular function (green).

To gain insight into the functional categorization of the assembled unigenes, GO classification was performed based on the NR annotation. A total of 69,973 unigenes could be assigned to GO terms, which were classified into 49 functional groups under three principle categories, i.e., Biological process, Molecular function and Cellular components (Figure 2). Under the Biological process category, “cellular process,” “metabolic process,” and “single-organism process” were predominantly represented. Within the Cellular components category, “cell,” “cell part,” and “organelle” were the most highly represented categories. For the Molecular function category, the most abundant of genes were associated with “binding” and “catalytic activity.”

**Figure 3:**
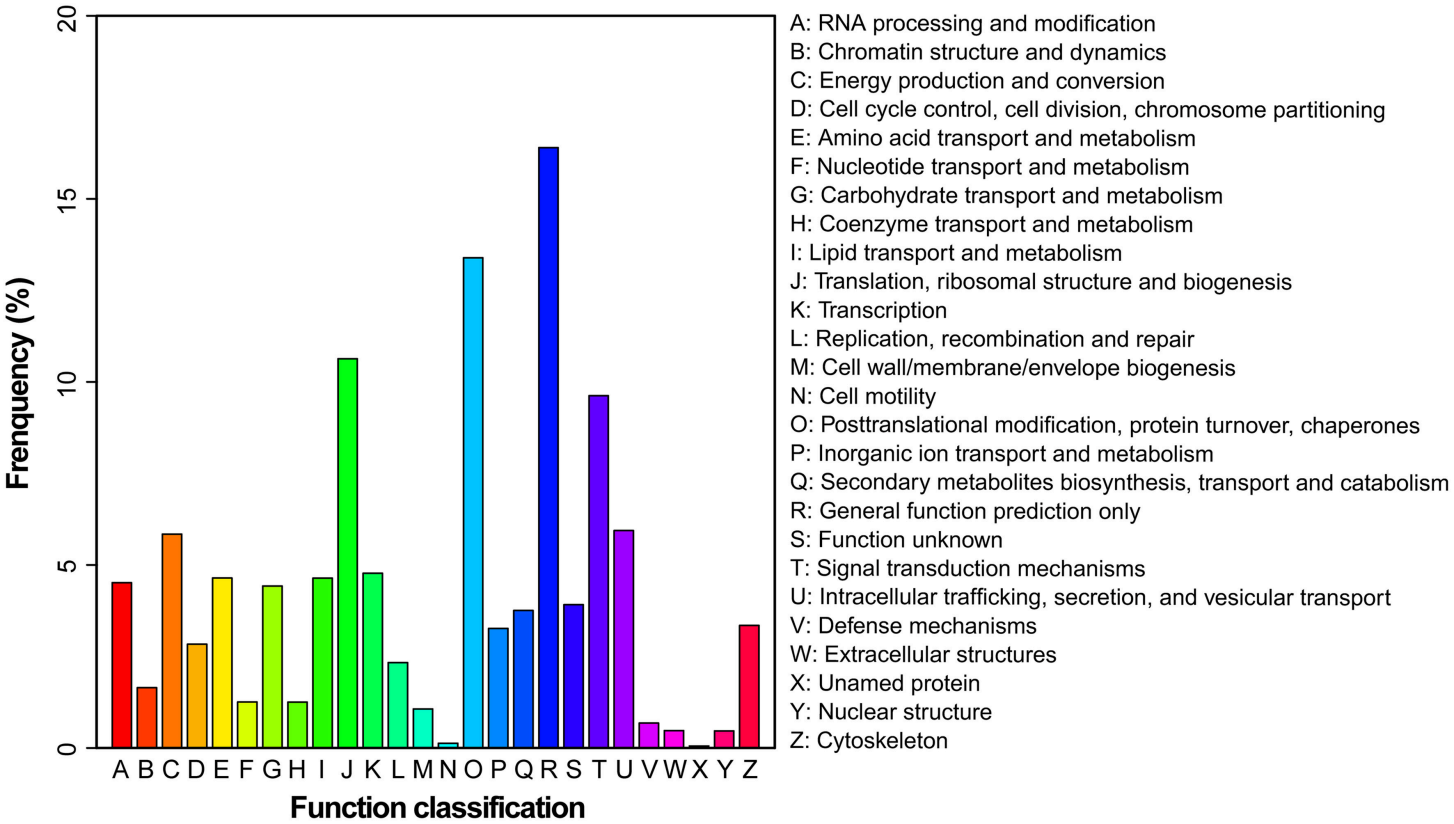
**Histogram presentation of KOG classification**. Unigenes with significant matches in the KOG database were classified into 26 categories.

To evaluate the effectiveness of the annotation and the completeness of the transcriptome library, all the assembled unigenes were subjected to search against the Eukaryotic Orthologous Groups (KOG) database. Based on sequence homology, unigenes were assigned into 26 KOG categories (Figure 3). Clusters “general function predicted only” and “posttranslational modification, protein turnover and chaperones” represented two of the largest ones, followed by “translation, ribosomal structure and biogenesis” and “signal transduction mechanisms.” In contrast, the clusters of “cell mobility” and “unnamed proteins” represented the smallest categories.

**Figure 4:**
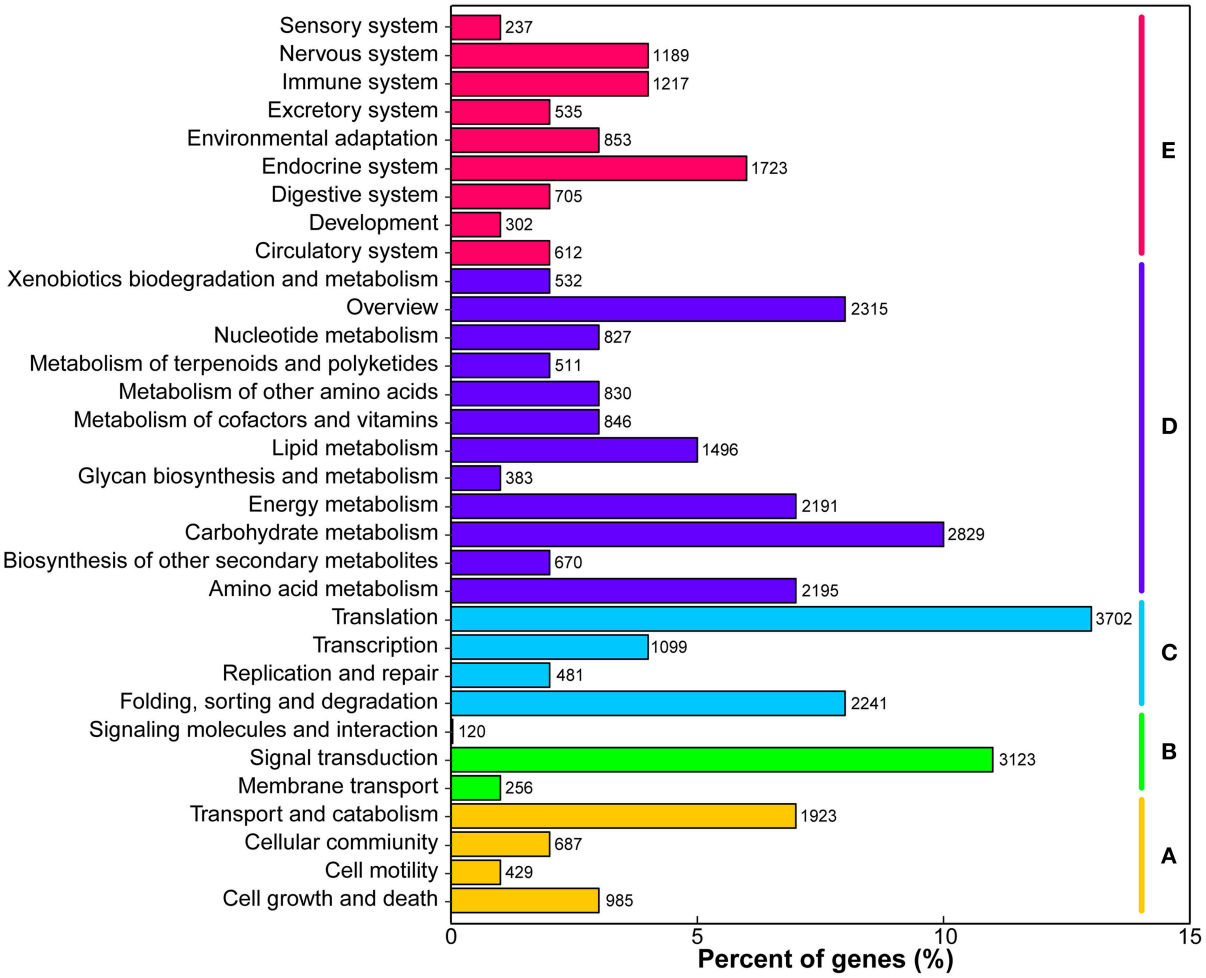
**Classification of unigenes in KEGG pathways**. The metabolism pathways are assigned into five categories. **(A)** Cellular processes. **(B)** Environmental information processing. **(C)** Genetic information processing. **(D)** Metabolism, and **(E)** Organismal systems.

KEGG pathway analysis was performed to identify the biochemical pathways in *M. lutarioriparius*. Based on sequence homology, a total of 34,668 (20.5%) unigenes were matched in the KEGG database and assigned to 32 pathways, covering five major KEGG categories (Figure 4). The three most represented pathways were “translation,” “signal transduction,” and “carbohydrate metabolism,” followed by “carbohydrate metabolism” and “overview,” whereas “signaling molecules and interaction” and “sensory system” pathways represented the smallest categories.

### Gene expression profiling

**Figure 5:**
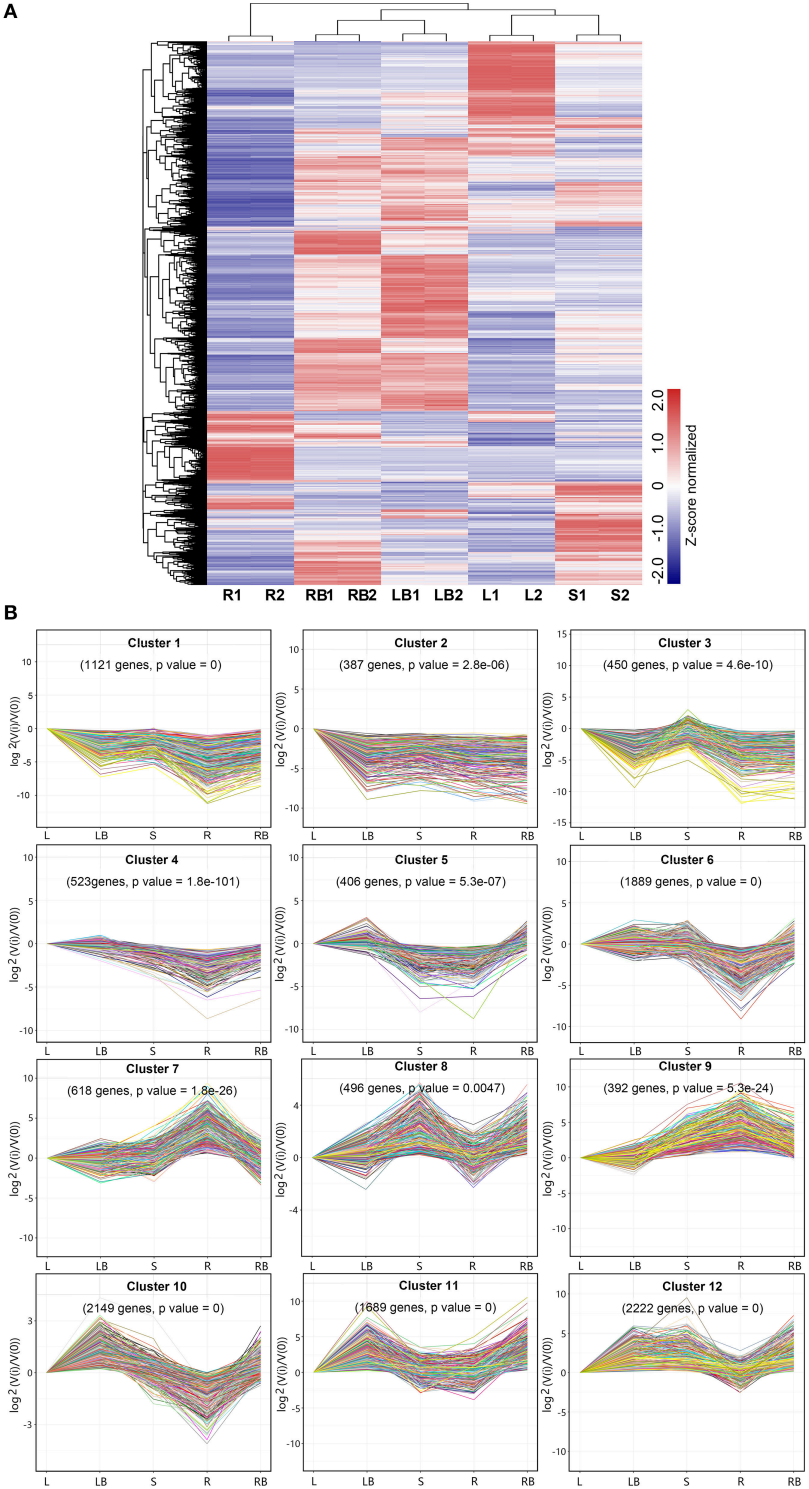
**Hierarchical clustering of differentially expressed genes and profile plots of clusters. (A)** Hierarchical clustering of differentially expressed genes across five different tissues based on Z-score normalized FPKM values. Blue color represents lower expressed genes, while red color represents higher expressed genes. **(B)** Expression profile plots of 12 significant clusters. L, leaf; S, stem; R, root; LB, lateral bud; RB, rhizome bud.

To gain a global view of gene expression pattern across different tissues, a heatmap was plotted using the Z-score normalized FPKM values (Figure 5A). All biological replicates were clustered together with almost identical expression profiles, indicating the reliability of sample collection and analytical procedure. In addition, the overall expression of rhizome buds and lateral buds samples correlated well compared to the correlation between the other samples, which suggest that these two tissues are much closely related (Figure S2).

To further investigate gene expression profiles across different tissues, K-means clustering was carried out to classify differentially expressed genes (Figure 5B). Totally, 12 clusters with distinctive expression patterns were identified. The most abundant group is cluster 12 comprised of 2,222 genes, whose expression was highest in lateral bud, stem and rhizome bud. The second largest group is cluster 10, which contained 1,889 genes, whose expression is much lower in root. In contrast, the collection of genes in cluster 7 and 9 mostly showed relatively higher expression in root. The collection of genes in cluster 1, 2, and 3 was predominantly expressed in leaf. Cluster 11 genes exhibited higher expression in both lateral bud and rhizome bud. These different gene expression profiles indicate that each tissue was associated with specific gene clusters.

### Tissue-enriched and differential expressed genes (DEGs)

**Figure 6:**
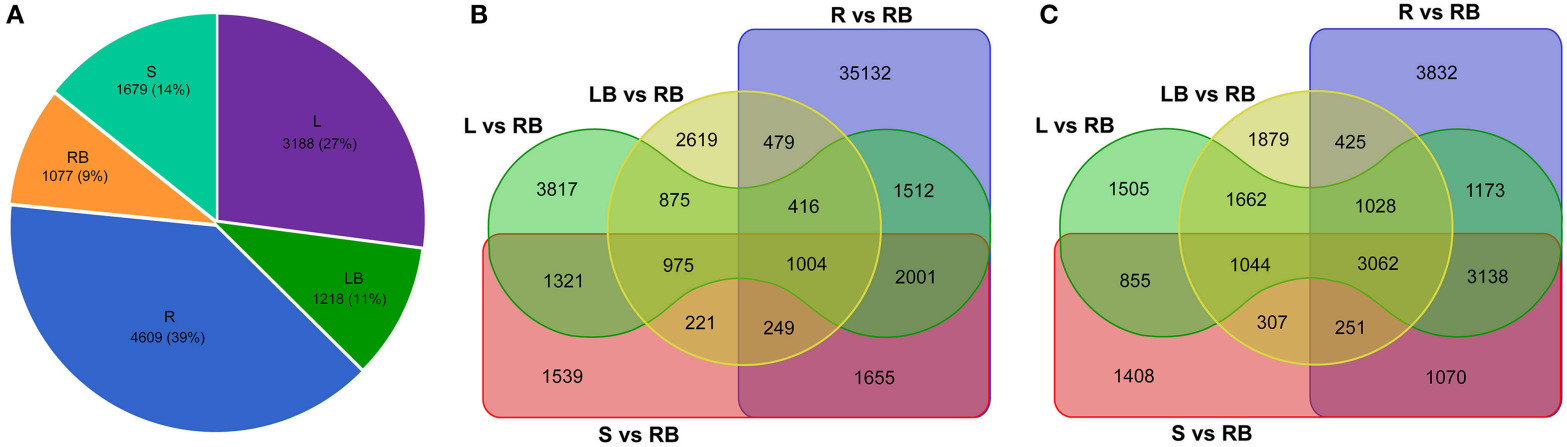
**Comparative analysis of tissue-enriched and differentially expressed genes. (A)** Pie chart showing the distribution of tissue-enriched genes in each tissue. **(B)** Venn diagram showing the up-regulated genes in tissues compared to rhizome bud. **(C)** Venn diagram showing the down-regulated genes in tissues compared to rhizome bud. L, leaf; S, stem; R, root; LB, lateral bud; RB, rhizome bud.

Knowledge of genes that are specifically or preferentially expressed in tissues can provide insights into the specialized developmental processes in these tissues. From this perspective, we sought to investigate the Differentially Expressed Genes (DEGs) in each tissue examined. All unigenes that have FPKM values higher than 10 and the fold change of relative expression is higher than 3.0 compared to other tissues were deemed as tissue-enriched genes. Totally, 4,609, 3,188, 1,679, 1,077, and 1,218 genes were identified to be specifically expressed in root, leaf, stem, rhizome bud and lateral bud, respectively (Figure 6A).

In order to identify genes specifically involved in the process of rhizome initiation and development, we further focused on the identification of DEGs in rhizome bud. As shown in the venn diagram of the distribution of DEGs in different tissues compared to rhizome bud (Figures 6B,C), root shared the largest number of differential expressed genes compared to rhizome bud with 42,443 genes up-regulated and 13,979 genes down-regulated, respectively. In contrast, lateral bud shared the smallest number of DEGs compared to rhizome bud, with 6,838 up-regulated and 9,658 down-regulated genes, respectively. In total, 1,004 common genes were up-regulated whereas 3,062 genes were down-regulated in the other four tissues compared to rhizome bud. These DGEs in rhizome buds may have important functional implications in rhizome development.

### GO and KEGG enrichment analysis

To gain insights into the functional categorization and metabolic pathways involved in rhizome development, DEGs in rhizome bud were subjected to enrichment analysis based on GO and KEGG pathways. Significantly enriched GO terms were found between rhizome bud and the other four tissues. Comparisons of these enriched GO terms in rhizome bud compared to leaf, lateral bud, stem and root revealed that the numbers and types of GO terms varied substantially (Figure S3). For example, “response to stimulus” and “DNA binding” were significantly enriched in rhizome bud compared to leaf, whereas “carbohydrate metabolic process” and “catalytic activity” were significantly enriched in a comparison between rhizome bud and stem. Nevertheless, several commonly enriched GO terms in rhizome bud compared to the other tissues, such as “cell,” “cell part,” “intracellular part” in Biological process category, “cellular process” and “metabolic process” in Cellular components category, and “binding,” and “catalytic activity” in Molecular functions category.

**Figure 7:**
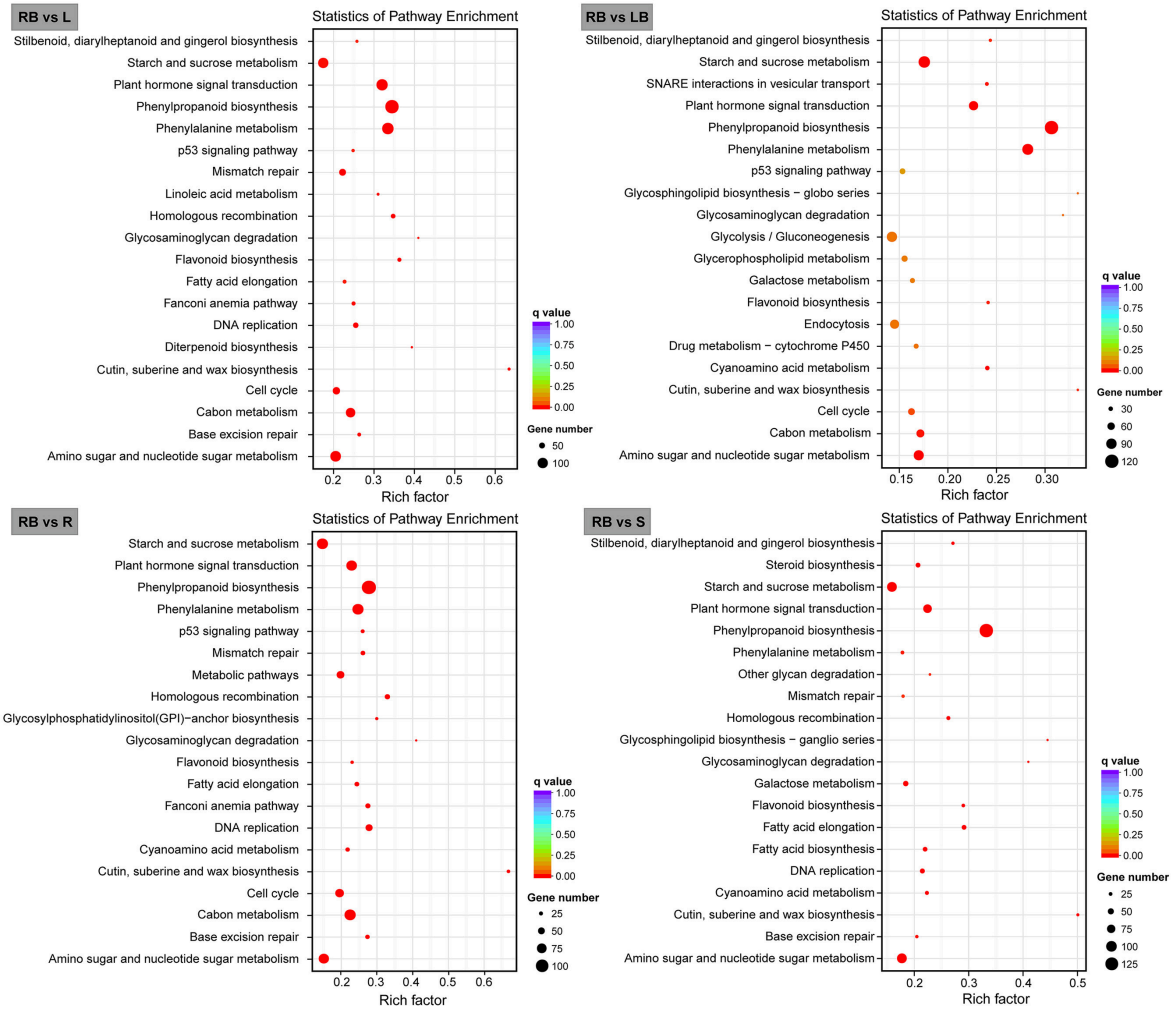
**Bar plots showing KEGG pathway enrichment of genes preferentially expressed in rhizome bud**. Top 20 KEGG pathways with the most significant enrichment for each tissue compared to rhizome bud are shown as bar plots. L, leaf; S, stem; R, root; LB, lateral bud; RB, rhizome bud.

We further mapped the DEGs in rhizome bud to the KEGG database and analyzed the enrichment of metabolic pathways. The top 20 enriched pathways in rhizome bud compared to the other tissues were listed in Figure 7. Among these pathways, a large number of genes were involved in the pathways related to “phenylpropanoid biosynthesis,” “starch and sucrose metabolism,” and “amino sugar and nucleotide sugar metabolism.” Remarkably, a number of genes were enriched in “plant hormone signal transduction” pathways in rhizome bud, suggesting that hormones may play crucial roles in the regulation of rhizome bud development.

### Identification of hormone signaling-related genes in rhizome development

**Figure 8:**
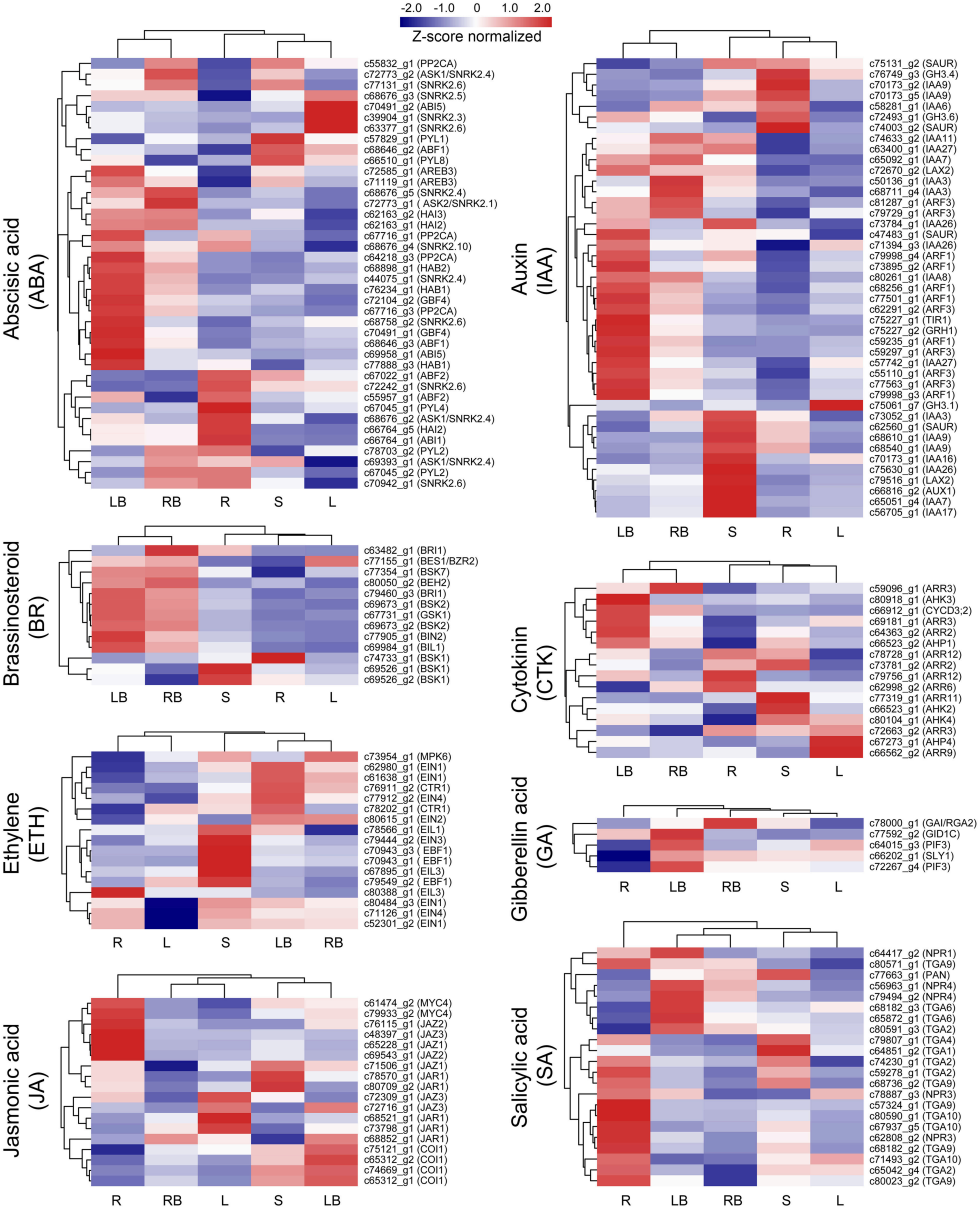
**Expression profile of hormone signaling-related genes across five different tissues**. The expression of genes involved in hormone signaling and transduction-related pathways was Z-score normalized and hierarchically clustered in heatmap. A color scale is shown at the top. Blue color indicates lower expression, while red color indicates higher expression. L, leaf; S, stem; R, root; LB, lateral bud; RB, rhizome bud.

Hormones have been implicated to paly crucial roles in diverse aspects of plant development processes. To gain insights into the functional roles of hormones during *M. lutarioriparius* rhizome development, we mapped the DGEs to hormone signaling and transduction pathways and analyzed their expression in different tissues (Figure 8). A total of 175 genes were identified to be associated with the biosynthesis, metabolism and signaling of eight hormones, including abscisic acid (ABA), auxin (IAA), ethylene (ETH), brassinosteroid (BR), jasmonic acid (JA), cytokinin (CTK), gibberellin acid (GA), and salicylic acid (SA). The genes associated with IAA biosynthesis and metabolism represented as the largest group with 43 members, followed by ABA signaling-related genes with 40 members. In contrast, the genes associated with GA signaling was the smallest group with only five members. All these hormone signaling and transduction-related genes exhibited differential expression across the different tissues examined. Noteworthy, a subset of 16 genes showed preferential expression in rhizome bud, suggesting a putative role of hormones signaling in the regulation of rhizome growth and development. Among these genes, seven genes were associated with IAA biosynthesis and metabolism, followed by six genes related to ABA, and two genes were involved in BR signaling (**Figure 10A** and Table S4).

### Identification of transcription factors in rhizome development

**Figure 9:**
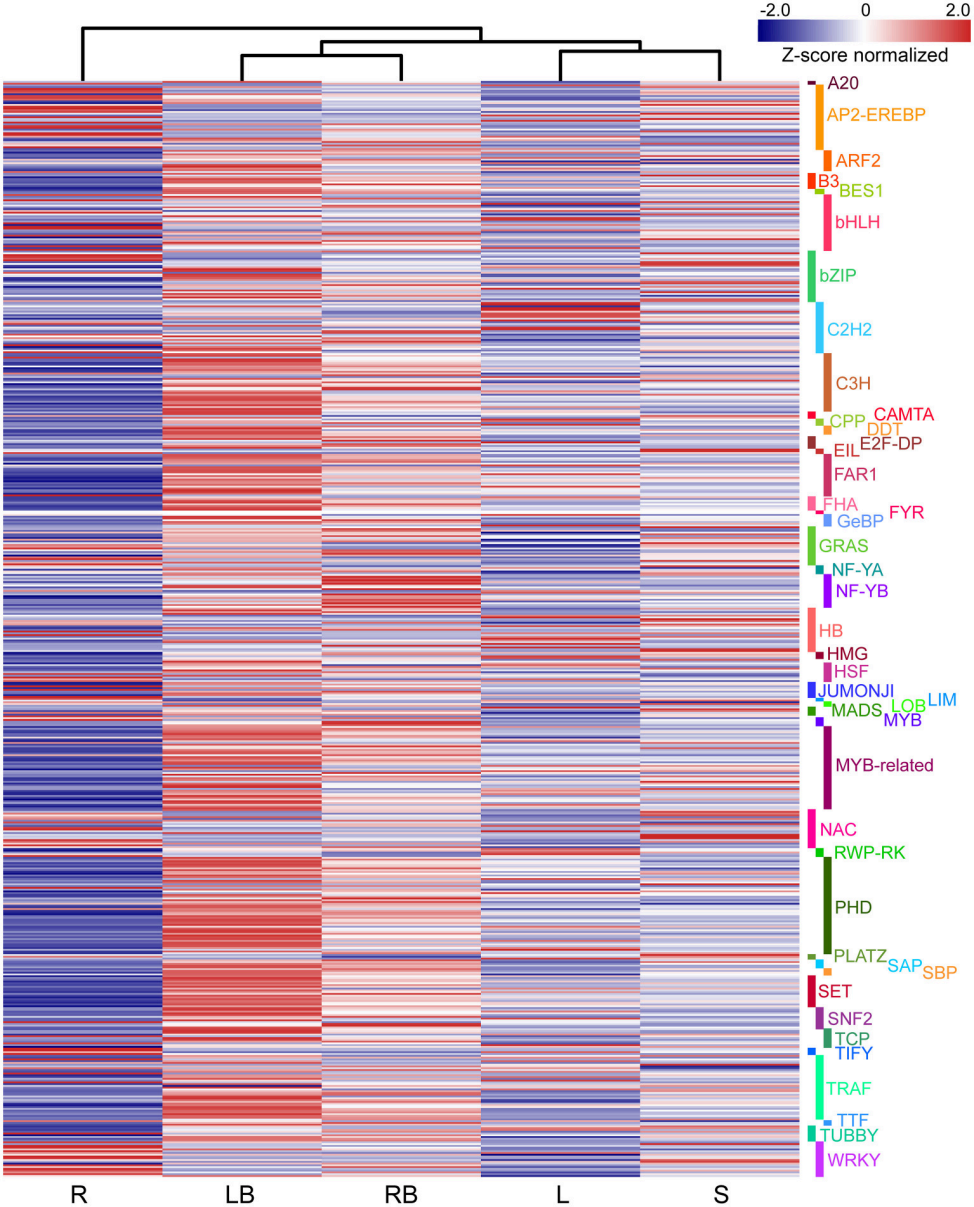
**The differential expression of TFs across five tissues**. The differential expression of TFs were depicted in heatmap based on Z-score normalized FPKM values. Blue color indicates lower expressed genes, while red color indicates higher expressed genes. L, leaf; S, stem; R, root; LB, lateral bud; RB, rhizome bud.

**Figure 10:**
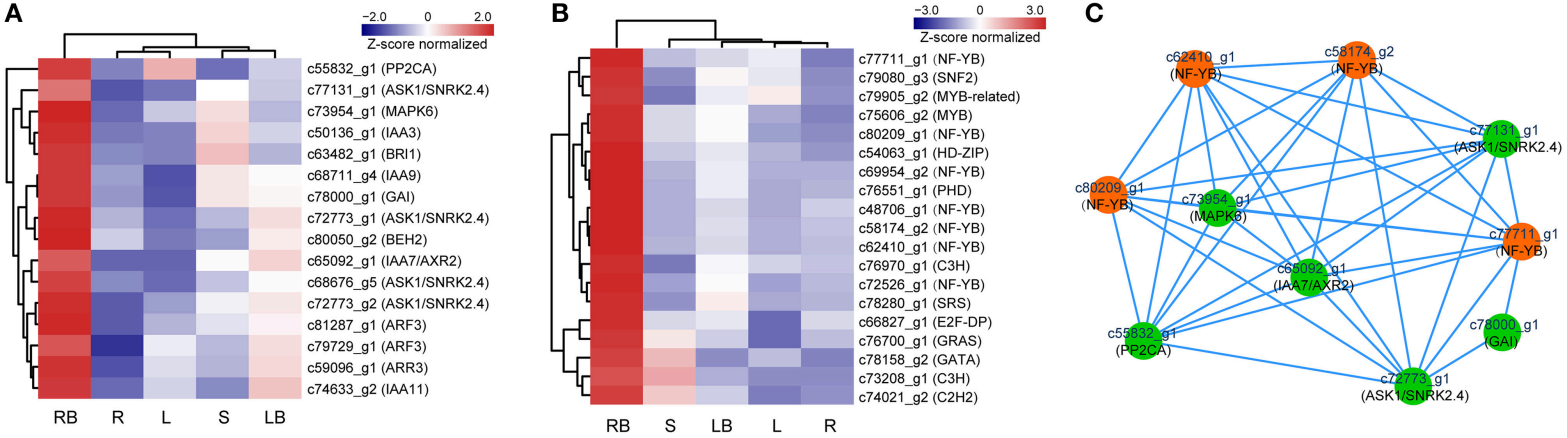
**Profiling and regulatory network of TFs and hormone signaling genes highly expressed in rhizome bud. (A)** Expression profile of 16 hormone signaling-related genes highly expressed in rhizome bud. **(B)** Hierarchical clustering of 19 TFs highly expressed in rhizome bud. **(C)** Predictive regulatory network among TFs and hormone signaling-related genes highly expressed in rhizome bud. The model was constructed based on gene co-expression and visualized by Cytoscape. TFs are in orange color and hormone signaling genes are in green color. L, leaf; S, stem; R, root; LB, lateral bud; RB, rhizome bud.

Accumulating evidence indicate that transcription factors (TFs) play critical roles in various plant development processes. To provide insights into the regulatory network underlying rhizome development, we examined the expression of TFs in different tissues especially their dynamic expression in rhizome bud. Totally, 690 TFs belonging to 44 different families were found to be differentially expressed in the tissues examined (Figure 9). The TFs displayed distinct expression patterns across the five tissues. A majority of TFs showed relatively broad expression patterns in all the tissues, while some exhibited distinctive tissue-specific patterns. Noticeably, 19 TFs were preferentially expressed in rhizome bud, implying that they might play important roles in the regulation of rhizome bud initiation and growth (Figure 10B and Table S4). These rhizome-specific TFs belong to different families with the largest members from NUCLEAR FACTOR Y (NF-Y) family. Furthermore, we constructed a putative regulation network based on gene co-expression patterns for the TFs and hormone signaling-related genes preferentially expressed in rhizome bud. The model indicated that four NF-YB TFs may putatively regulate the expression of six hormone signaling genes related to ABA and IAA. (Figure 10C).

### Validation of the expression of DEGs by qRT-PCR

To validate the reliability of the expression profiling obtained by RNA-seq, 24 genes with differential expression in rhizome bud were selected for quantitative real-time RT-PCR (qRT-PCR) analysis. The genes chosen for qRT-PCR analysis included 12 TFs and 12 hormone signaling-related genes. For all these genes, the results of qRT-PCR exhibited almost identical expression patterns as compared to the transcriptome profiling results. In addition, good agreement between qRT-PCR and RNA-seq results was also supported by a significant positive correlation between them as revealed by Pearson correlation analysis (*R*^2^ = 0.9219, *P* < 0.05) (Figure S4). These results verified the accuracy and reliability of the RNA-Seq analysis.

## Discussion

As a promising lignocellulosic bioenergy plant, one of the superior characteristics of *M. lutarioriparius* is its vigorous rhizome as compared with the other Miscanthus species (e.g., *M. sacchariflorus* and *M. sinensis*). Rhizomatousness is an ideal trait for *M. lutarioriparius* to rapidly set up canopy and exert superior stress tolerances especially when cultivated in marginal land. In addition, the *M. lutarioriparius* rhizome also serves as the primary propagation organ as well as the main storage organ for reservoirs of nutrients in fall season. Thus, understanding of the biological processes involved in rhizome development and growth is of fundamental importance. In this study, we provided the first *de novo* transcriptome data from five tissues of *M. lutarioriparius* with the main objective to identify genes putatively involved in rhizome bud development.

By transcriptome profiling analysis, 3,062 genes were identified to be preferentially expressed in *M. lutarioriparius* rhizome bud (Figure 6C). KEGG enrichment analysis revealed that a large number of genes were involved in the pathways related to “phenylpropanoid biosynthesis,” “starch and sucrose metabolism,” “amino sugar and nucleotide sugar metabolism,” and “hormone signaling and transduction” (Figure 7). These pathways are principally involved in cell wall biosynthesis, cell proliferation, nutrient accumulation, primary metabolism and hormone signaling. The enrichment of these pathways in rhizome is consistent with the physiological roles of *M. lutarioriparius* rhizome in asexual propagation and serves as the storage organ of carbohydrate metabolisms in fall season. Transcriptome analysis has already been carried out in several rhizomatous species (e.g., reed, lotus, bamboo and sorghum) including two Miscanthus species (*M*. × *gigantues* and *M. sacchariflorus*) to identify candidate genes involved in rhizome development (Jang et al., 2006; Wang et al., 2010; He et al., 2012; Barling et al., 2013; Cheng et al., 2013; Kim et al., 2014; Zhang et al., 2014; Yang et al., 2015). A large number of highly expressed genes in rhizomes have been reported to be predominantly associated with “primary metabolism,” and “hormone signaling and stress response” etc. (Jang et al., 2006; Wang et al., 2010; He et al., 2012; Cheng et al., 2013; Yang et al., 2015). However, we did not find significant enrichment of pathways involved in response to stresses in *M. lutarioriparius* rhizome in the current study. This may be partly attributed to the situation that the *M. lutarioriparius* plants we used for this study were maintained in optimized growth conditions and irrigated regularly. Similar results were also reported for *M*. × *giganteus* transcriptome, in which genes involved in “hormone signaling” pathways were significantly enriched in spring rhizomes, whereas genes associated with “amino acid metabolism and seed maturation” pathways were much higher represented in fall rhizomes (Barling et al., 2013). Moreover, the highly expressed genes in rhizomes of several herbal plants such as Cangzhu (*A. lancea*), Ginger (*Z. officinale*), and turmeric (*C. longa*) were associated with pathways in secondary metabolisms (Koo et al., 2013; Huang et al., 2016). This is not unexpected considering the fact that rhizomes are the main storage organs for abundant amounts of secondary metabolisms in these rhizomatous herbal plants.

Although substantial progresses have been gained in our understanding of biological processes in rhizome development, the molecular mechanisms underlying rhizome development still remain largely unknown. However, as rhizome and tiller are originally derived from the axillary meristems from the lowermost part of the shoot, it can be assumed that genes controlling tiller development should also play a role in rhizome development and growth. This assumption is supported by the comparative transcriptome analysis of lateral bud and rhizome bud in this study. Among the five different tissues analyzed, lateral bud and rhizome bud shared the highest overall correlation coefficient in expression (Figure 5 and Figure S3). Correspondingly, these two tissues also shared the least number of DGEs compared to the comparisons among the other tissues (Figures 6B,C). These results suggested that a certain percent of genes might play largely overlapped roles in governing the morphogenesis and development of rhizome bud and lateral bud in *M. lutarioriparius*. However, their definitive roles awaits further functional characterization by transgenic studies.

TFs play important roles in various plant developmental processes. In the last two decades, studies especially in model species *Arabidopsis* and rice have revealed that a substantial numbers of TFs play crucial role in the regulation of tiller initiation and plant architecture. For example, *TEOSINTE BRANCHED 1* (*TB1*) encoding a TCP domain transcription factor was originally identified as a negative player in regulating axillary bud outgrowth in maize (Doebley et al., 1997). The homologs of *TB1* were also identified in other plant species including *Arabidopsis* (Aguilar-Martínez et al., 2007), rice (Takeda et al., 2003), and sorghum (Kebrom et al., 2006). *REVOLUTA* (*REV*), a member of HD-ZIP III TF gene family, is required for the initiation of axillary meristem in *Arabidopsis* (Talbert et al., 1995; Otsuga et al., 2001). *LATERAL SUPPRESSOR* (*LAS*) encoding a GRAS family transcription factor specifically regulates the initiation of axillary meristem in *Arabidopsis* (Greb et al., 2003). *MONOCULM1* (*MOC1*), an ortholog of *LAS* in rice, is a key player involved in axillary bud initiation and tiller outgrowth (Li et al., 2003). Although a substantial set of genes involved in tiller development have been characterized and the underlying molecular mechanisms largely elucidated, it still remains to be answered to what degree does the molecular mechanism controlling tiller development relevant to that of rhizome growth. Interestingly, our transcriptome profiling revealed that a subset of TFs belonging to NF-YB, MYB and GRAS families were predominantly expressed in rhizome bud (Figure 10B). However, no homologs to these above mentioned TFs except for one GRAS family member were discovered in our analysis. We further analyzed the TFs that were specifically expressed in lateral bud. The results showed that several TFs homologous to *TB1, REV*, and *LAS* were preferentially expressed in lateral bud (Figure 9). These results indicated that the rhizome development of *M. lutarioriparius* is substantially different from that of tiller development.

In addition, accumulating evidence indicate that hormones affect diverse aspects of plant development processes, especially in the determination of tiller outgrowth and the formation of storage organs. For example, exogenous application of GA can significantly promote rhizome elongation in two rhizomatous grass species (Ma and Huang, 2016). In addition, genes associated with GA signaling pathways are enriched in rhizomes in several transcriptome analysis (Hu et al., 2011; Cheng et al., 2013). CTK plays important roles in the regulation of axillary bud initiation and outgrowth (Choi and Hwang, 2007), and exogenous application of CTK can stimulate rhizome formation in tall fescue (*Festuca arundinacea*) (Ma and Huang, 2016). IAA and CTK are implicated to act antagonistically in the regulation of axillary buds growth. Apically derived auxin inhibits the lateral bud outgrowth, whereas CTK relieves the inhibition. It is the homeostatic balance of their levels that determinates the initiation and outgrowth of axillary buds (Shimizu-Sato et al., 2009). In this study, 16 genes associated with ABA, IAA, BR, and GA signaling and transduction were preferentially expressed in rhizome bud, implicating the putative roles of these hormones in rhizome development in *M. lutarioriparius*. Furthermore, these hormone signaling genes were predicted to constitute a complicated regulation network with four NF-YB TFs identified in this study (Figure 10C). These genes identified provided excellent candidates for further functional characterization toward unraveling the molecular mechanisms underlying rhizome development in *Miscanthus* species.

## Conclusions

In this study, a *de novo* assembly of transcriptome data from five tissues of *M. lutarioriparius* was performed with the main objective to provide preliminary insights into rhizome development. The comparative transcriptome analysis revealed differential and tissue-enriched genes, which were enriched in KEGG pathways associated with primary metabolism and hormone signaling. Noteworthy, 19 TFs and 16 hormone signaling and transduction-related genes were identified to be preferentially expressed in rhizome bud, highlighting the involvement of TFs and hormones in the regulation of rhizome development. Taken together, the transcriptome dataset presented here identified a subset of candidate genes putatively associated with rhizome formation and growth in *M. lutarioriparius*, thus laid a foundation for further functional genomics studies on rhizome development in *Miscanthus* species.

## Author contributions

RH designed the experiment, performed data processing and drafted the manuscript. CY and GH helped in bioinformatics analysis and data interpretation. XW, SP, and KH prepared the materials and performed the experiments. CJ participated in the design of the study, helped in data processing, and revision of the manuscript. YK assisted in results interpretation and manuscript preparation. GZ conceived the study and revised the manuscript. All authors read and approved the final version of the manuscript.

### Conflict of interest statement

The authors declare that the research was conducted in the absence of any commercial or financial relationships that could be construed as a potential conflict of interest.

## Supplementary material

Figure S1 **Length distribution of assembled transcripts and unigenes**.

Figure S2 **GO enrichment of genes differentially expressed in rhizome bud compared to each of the other four tissues**.

Figure S3 **Pearson's correlation coefficient among different tissues**.

Figure S4 **Validation of expression between transcriptome profiling and qRT-PCR analysis for 24 genes**.

Table S1 **Primer sequences used in qRT-PCR analysis**.

Table S2 **Sequencing statistics of the transcriptome from five tissues**.

Table S3 **Annotation statistics of unigenes in publicly available databases**.

Table S4 **FPKM values of TFs and hormone signaling-related genes in five tissues**.

### Availability of supporting data

All the sequencing data used in this research are available from the NCBI Sequence Read Archive (SRA) database with accession number SRP094875.
