# Supplementary figures and images for: De novo Transcriptome Analysis of Miscanthus lutarioriparius Identifies Candidate Genes in Rhizome Development
Source: Front Plant Sci. 2017 Apr 12;8:492. doi: 10.3389/fpls.2017.00492 (PMC5388781; doi:10.3389/fpls.2017.00492)

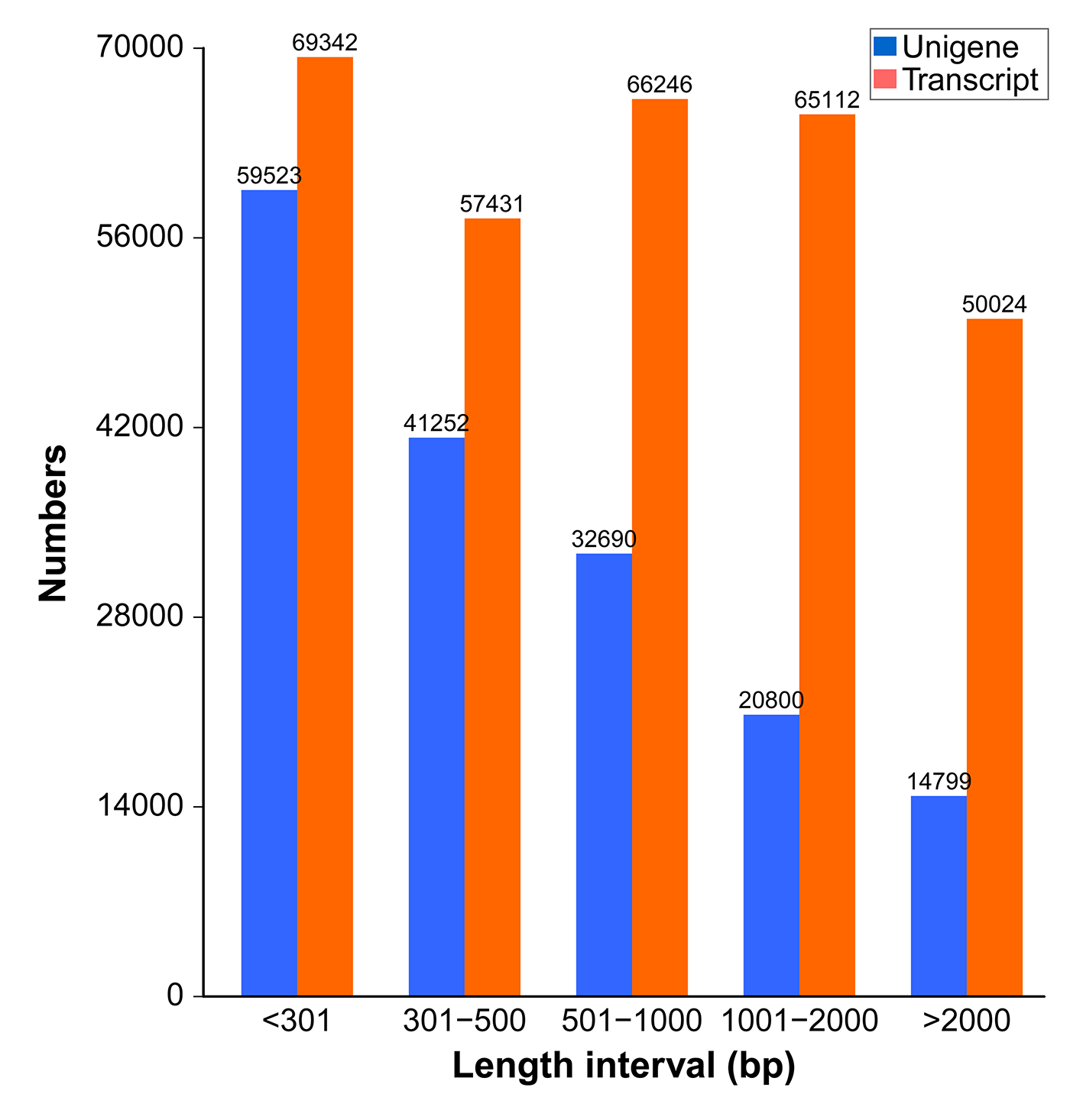

Supplement: Figure S1 — Length distribution of assembled transcripts and unigenes. [file Image1.TIF]

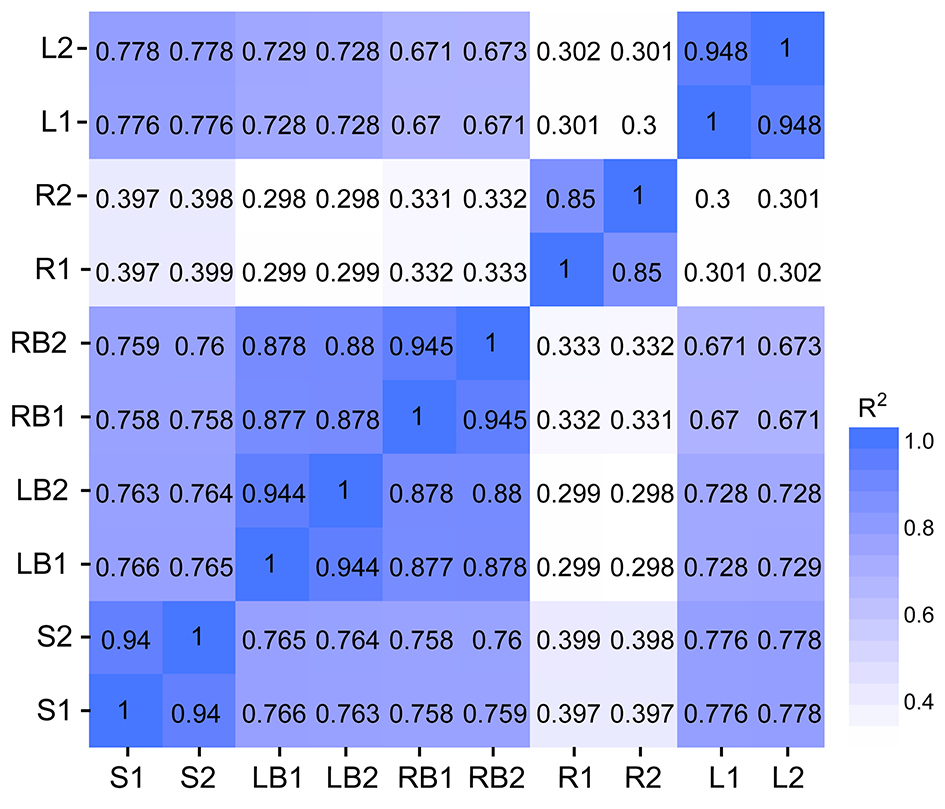

Supplement: Figure S2 — GO enrichment of genes differentially expressed in rhizome bud compared to each of the other four tissues. [file Image2.TIF]

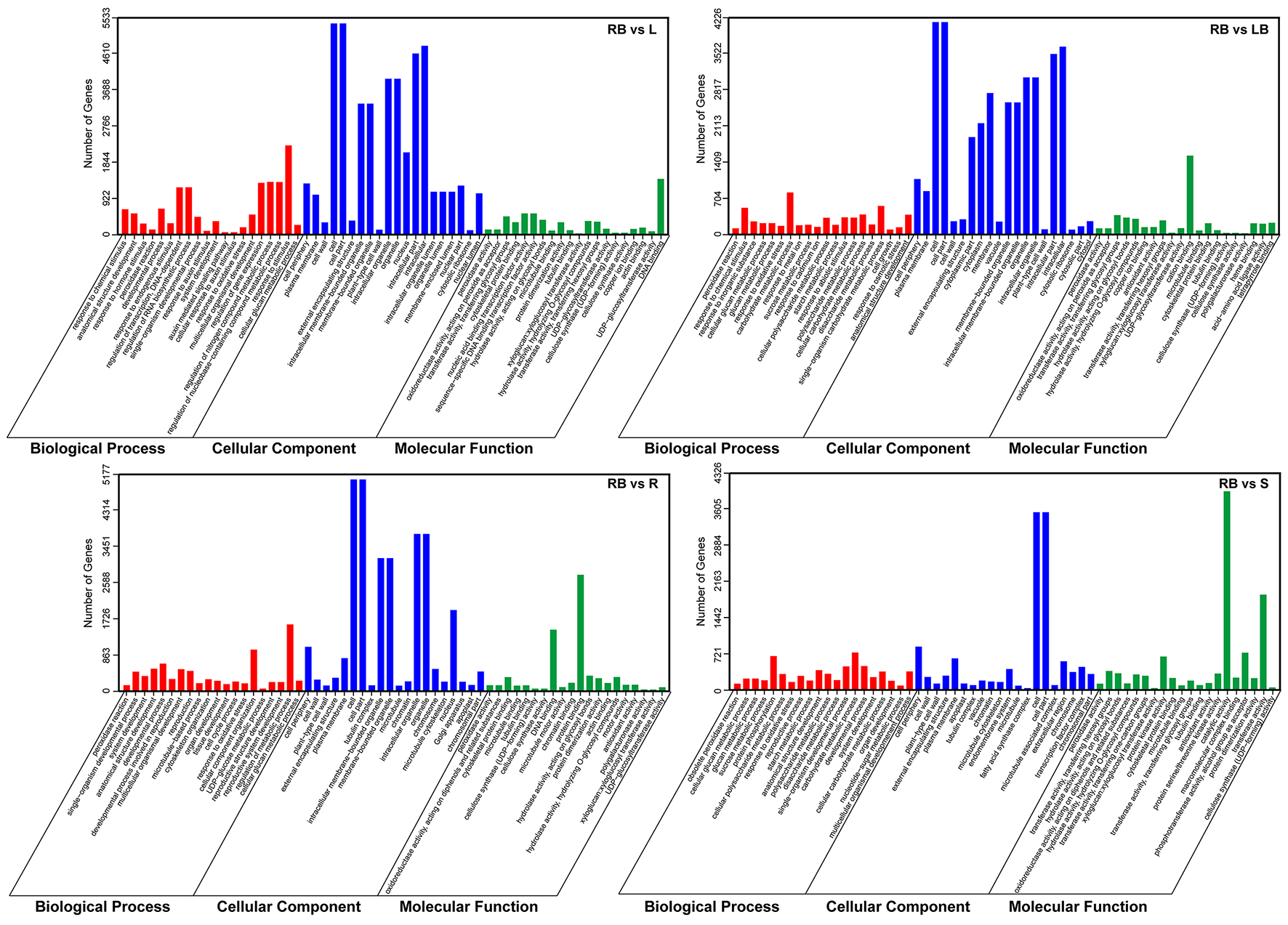

Supplement: Figure S3 — Pearson's correlation coefficient among different tissues. [file Image3.TIF]

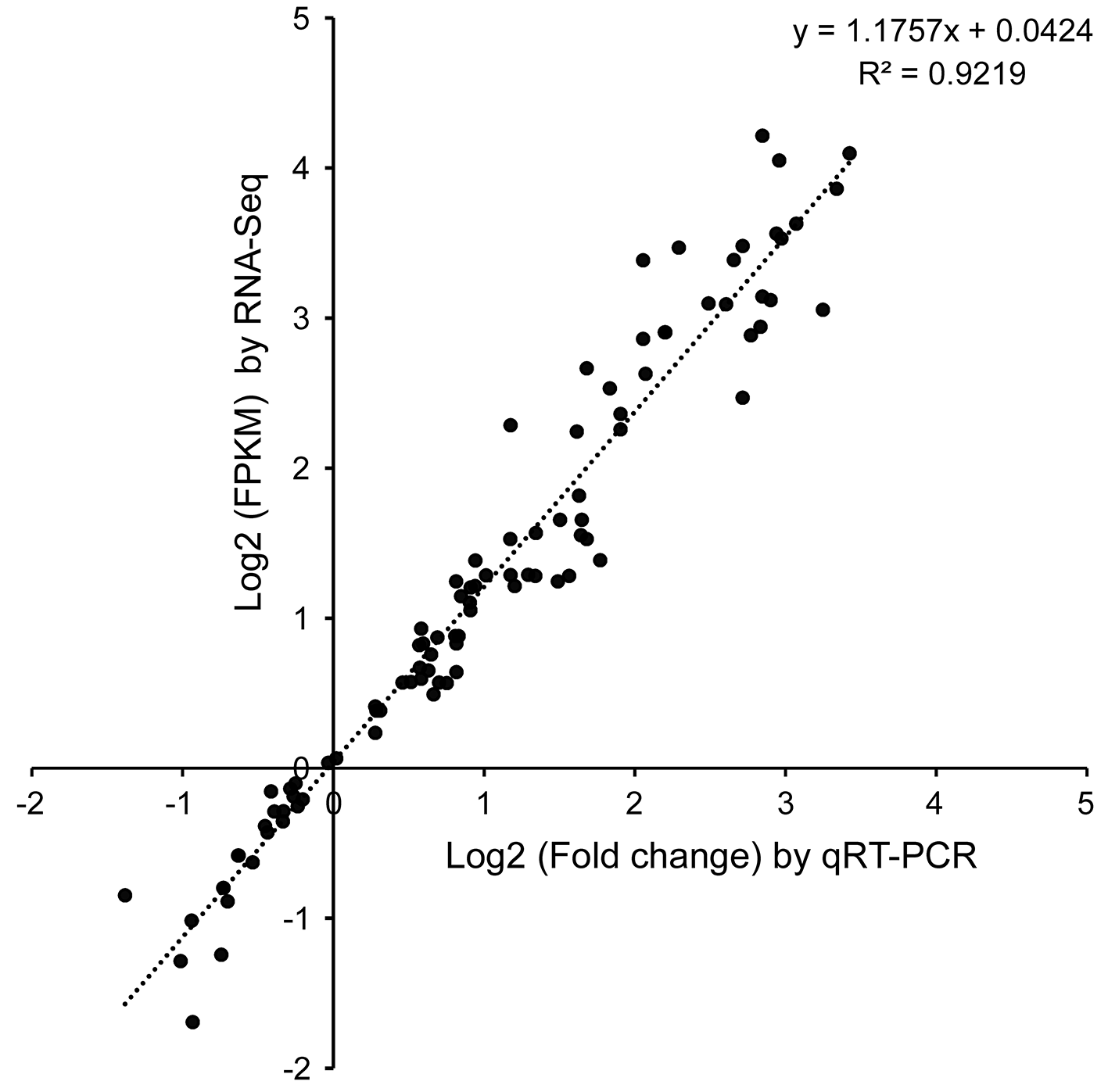

Supplement: Figure S4 — Validation of expression between transcriptome profiling and qRT-PCR analysis for 24 genes. [file Image4.TIF]
