# Supplementary material for: De novo Transcriptome Analysis of Miscanthus lutarioriparius Identifies Candidate Genes in Rhizome Development
Source: Front Plant Sci. 2017 Apr 12;8:492. doi: 10.3389/fpls.2017.00492 (PMC5388781; doi:10.3389/fpls.2017.00492)
Supplement: Table S1 — Primer sequences used in qRT-PCR analysis. [file Table1.DOCX]

**Table S1.** Primer sequences used in qRT-PCR analysis.

| **Primer** | **Primer sequence (5’→3’)** | **Amplicon length (bp)** |
| --- | --- | --- |
| c55832_g1_F | CTTATTATGCTATTGATGGATGT | 90 |
| c55832_g1_R | GAGACTCATAGTATCTATCTACAA |  |
| c65092_g1_F | GACTTGAACACTGACTGA | 164 |
| c65092_g1_R | AGAAGAACCAATCTCCATAG |  |
| c73954_g1_F | TGCTATTCATTGTGTTCTTG | 177 |
| c73954_g1_R | ATTCACTCCACTACATTCC |  |
| c72773_g2_F | GTAACGACTAGACGCTAAT | 113 |
| c72773_g2_R | GTGTTCTCTTCTGCTCAT |  |
| c78000_g1_F | CCTTATCTGTTCTAGTGATGA | 81 |
| c78000_g1_R | CTATTCGTCCTCTATATGATGA |  |
| c77131_g1_F | AACATATTGGAGAGTGGATTA | 195 |
| c77131_g1_R | AACAGTGAATAGAGGATACG |  |
| c62410_g1_F | CTAGGGCTTTGTTTCACG | 81 |
| c62410_g1_R | CTAGGGCTTTGTTTCACC |  |
| c58174_g2_F | TGCGCTTCGAGTGGCTTC | 78 |
| c58174_g2_R | AGCAGGAGACCTCGGAAT |  |
| c80209_g1_F | TAGGAGTGTATGGAATGTTC | 104 |
| c80209_g1_R | CTAGTCAGTAGTGTCTATTGG |  |
| c77711_g1_F | GTGGAGACCTACAAGATC | 106 |
| c77711_g1_R | TGTCATTGATGAAGGAGTT |  |
| c76551_g1_F | GCAATACAATCCAGAATCC | 93 |
| c76551_g1_R | GACCATCAATAAGAGAATAGC |  |
| c48706_g1_F | GCAAGAGAGAGGAAATCA | 86 |
| c48706_g1_R | GTGGGATGTAAACAAACG |  |
| c79905_g2_F | CAGTCCACTCTTCAATCA | 103 |
| c79905_g2_R | CTCCGTTCCTTATCTTCAA |  |
| c75606_g2_F | TAGCACTAAGGTCAATATAACT | 193 |
| c75606_g2_R | GAATACTCTGTCGGTAGC |  |
| c76970_g1_F | CCAACCTTGAAGAGATGTA | 78 |
| c76970_g1_R | AGTAGACTGAGTGACCTT |  |
| c81287_g1_F | ATAACTCGCTTGGAATATGA | 104 |
| c81287_g1_R | AATGCTTGATTGCTTCTTC |  |
| c69954_g2_F | GAAGTGGCGAATCTATGT | 121 |
| c69954_g2_R | GCAATCAGAATCAGAATGG |  |
| c79729_g1_F | GAGTGCTAGGGTTTTAGG | 99 |
| c79729_g1_R | GTTCTCATGTTCCTCCTC |  |
| c66827_g1_F | GGCTCATCATCAAGTCAT | 92 |
| c66827_g1_R | CCTAACAAGAACCTTCCTAT |  |
| c68711_g4_F | GCTGTTCAATGTTCATCC | 124 |
| c68711_g4_R | CTGCGTTCTACTCCTAAG |  |
| c63482_g1_F | CACGACAATAATTCTGACAA | 85 |
| c63482_g1_R | GAAGACATAAGAGCGGTAT |  |
| c74633_g2_F | ATGCTTGTTGGAGATGTT | 100 |
| c74633_g2_R | GAAGGAACTAACCGAGAC |  |
| c80050_g2_F | GAATCTAGCCAATCCAGTT | 130 |
| c80050_g2_R | GAGTAGAGTGTTGTGTCAT |  |
| c59096_g1_F | TCAGTACAGTCAGTCAGT | 137 |
| c59096_g1_R | GATACAGTAGCAGCAGAC |  |
| *ACTIN11*-F | CTCGTCTTCCTCACCGTTATCAC | 199 |
| *ACTIN11*-R | GCGTCATCTCCAGCGAACC |  |
