# Supplementary material for: De novo Transcriptome Analysis of Miscanthus lutarioriparius Identifies Candidate Genes in Rhizome Development
Source: Front Plant Sci. 2017 Apr 12;8:492. doi: 10.3389/fpls.2017.00492 (PMC5388781; doi:10.3389/fpls.2017.00492)
Supplement: Table S2 — Sequencing statistics of the transcriptome from five tissues. [file Table2.DOCX]

**Table S2.** Sequencing statistics of the transcriptome from five tissues.

| **Sample** | **Raw Reads** | **Clean Reads** | **Clean Bases (GB)** | **Error**  **(%)** | **Q20**  **(%)** | **Q30**  **(%)** | **GC Content**  **(%)** |
| --- | --- | --- | --- | --- | --- | --- | --- |
| L1_1 | 28293433 | 25398160 | 3.17G | 0.03 | 96.41 | 92.65 | 53.55 |
| L1_2 | 28293433 | 25398160 | 3.17G | 0.04 | 93.57 | 87.97 | 53.51 |
| L2_1 | 30913599 | 27743351 | 3.47G | 0.03 | 96.38 | 92.60 | 53.58 |
| L2_2 | 30913599 | 27743351 | 3.47G | 0.04 | 93.72 | 88.20 | 53.54 |
| LB1_1 | 30583605 | 28907879 | 3.61G | 0.04 | 95.36 | 90.62 | 51.16 |
| LB1_2 | 30583605 | 28907879 | 3.61G | 0.04 | 92.56 | 86.15 | 51.10 |
| LB2_1 | 31256399 | 29626495 | 3.70G | 0.04 | 95.47 | 90.84 | 51.93 |
| LB2_2 | 31256399 | 29626495 | 3.70G | 0.04 | 92.55 | 86.13 | 51.87 |
| S1_1 | 27829749 | 26375800 | 3.29G | 0.03 | 95.59 | 91.02 | 52.16 |
| S1_2 | 27829749 | 26375800 | 3.29G | 0.04 | 93.92 | 88.31 | 52.15 |
| S2_1 | 28299223 | 26811099 | 3.35G | 0.04 | 95.54 | 90.89 | 52.50 |
| S2_2 | 28299223 | 26811099 | 3.35G | 0.04 | 93.69 | 87.80 | 52.50 |
| RB1_1 | 26877553 | 25958731 | 3.24G | 0.03 | 96.31 | 92.48 | 52.55 |
| RB1_2 | 26877553 | 25958731 | 3.24G | 0.04 | 93.39 | 87.69 | 52.48 |
| RB2_1 | 25305252 | 24293221 | 3.04G | 0.03 | 96.38 | 92.60 | 52.57 |
| RB2_2 | 25305252 | 24293221 | 3.04G | 0.04 | 93.48 | 87.83 | 52.51 |
| R1_1 | 25577126 | 24069879 | 3.01G | 0.03 | 96.32 | 92.54 | 51.55 |
| R1_2 | 25577126 | 24069879 | 3.01G | 0.04 | 93.88 | 88.54 | 51.53 |
| R2_1 | 27444181 | 25923982 | 3.24G | 0.03 | 96.19 | 92.31 | 51.38 |
| R2_2 | 27444181 | 25923982 | 3.24G | 0.04 | 94.03 | 88.77 | 51.37 |
