# Supplementary material for: De novo Transcriptome Analysis of Miscanthus lutarioriparius Identifies Candidate Genes in Rhizome Development
Source: Front Plant Sci. 2017 Apr 12;8:492. doi: 10.3389/fpls.2017.00492 (PMC5388781; doi:10.3389/fpls.2017.00492)
Supplement: Table S3 — Annotation statistics of unigenes in publicly available databases. [file Table3.DOCX]

**Table S3.** Annotation statistics of unigenes in publicly available databases.

| **Annotation in database** | **Number of unigenes** | **Percentage (%)** |
| --- | --- | --- |
| Annotated in NR | 85821 | 50.76 |
| Annotated in NT | 55811 | 33.01 |
| Annotated in KO | 29471 | 17.43 |
| Annotated in SwissProt | 54851 | 32.44 |
| Annotated in PFAM | 60203 | 35.6 |
| Annotated in GO | 66973 | 39.61 |
| Annotated in KOG | 34668 | 20.5 |
| Annotated in all Databases | 10249 | 6.06 |
| Annotated in at least one Database | 103899 | 61.45 |
| Total unigenes | 169064 | 100 |
